# *N*-Benzoyl-2-methyl­benzene­sulfonamide

**DOI:** 10.1107/S1600536810012067

**Published:** 2010-04-02

**Authors:** P. A. Suchetan, B. Thimme Gowda, Sabine Foro, Hartmut Fuess

**Affiliations:** a Department of Chemistry, Mangalore University, Mangalagangotri 574 199, Mangalore, India; b Institute of Materials Science, Darmstadt University of Technology, Petersenstrasse 23, D-64287 Darmstadt, Germany

## Abstract

In the title compound, C_14_H_13_NO_3_S, the conformation of the N—H bond in the C—SO_2_—NH—C(O) segment is *anti* to the C=O bond. The tolyl and benzoyl groups are twisted about the S—N bond, with a C—S—N—C torsion angle of 68.8 (4)°. The dihedral angle between the sulfonyl and the benzoyl benzene rings is 73.9 (1)°. In the crystal, the mol­ecules are linked into *C*(4) chains along the *c* axis by N—H⋯O hydrogen bonds.

## Related literature

For background literature and similar structures, see: Gowda *et al.* (2009 ▶, 2010 ▶); Suchetan *et al.* (2010 ▶).
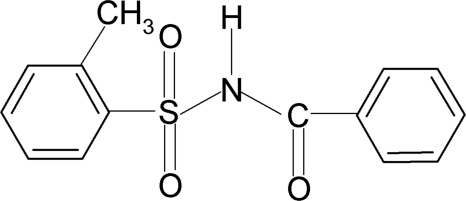


## Experimental

### 

#### Crystal data

C_14_H_13_NO_3_S
*M* *_r_* = 275.31
Orthorhombic, 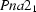

*a* = 19.772 (2) Å
*b* = 11.894 (1) Å
*c* = 5.6368 (5) Å
*V* = 1325.6 (2) Å^3^
*Z* = 4
Mo *K*α radiation
μ = 0.25 mm^−1^
*T* = 299 K
0.30 × 0.18 × 0.04 mm

#### Data collection

Oxford Diffraction Xcalibur diffractometer with a Sapphire CCD detector
Absorption correction: multi-scan (*CrysAlis RED*; Oxford Diffraction, 2009 ▶) *T*_min_ = 0.930, *T*_max_ = 0.990
4891 measured reflections
2028 independent reflections
1760 reflections with *I* > 2σ(*I*)
*R*_int_ = 0.029

#### Refinement

*R*[*F* ^2^ > 2σ(*F* ^2^)] = 0.053
*wR*(*F* ^2^) = 0.132
*S* = 1.18
2028 reflections
176 parameters
2 restraints
H atoms treated by a mixture of independent and constrained refinement
Δρ_max_ = 0.52 e Å^−3^
Δρ_min_ = −0.38 e Å^−3^
Absolute structure: Flack (1983 ▶), 541 Friedel pairs
Flack parameter: −0.11 (15)

### 

Data collection: *CrysAlis CCD* (Oxford Diffraction, 2009 ▶); cell refinement: *CrysAlis RED* (Oxford Diffraction, 2009 ▶); data reduction: *CrysAlis RED*; program(s) used to solve structure: *SHELXS97* (Sheldrick, 2008 ▶); program(s) used to refine structure: *SHELXL97* (Sheldrick, 2008 ▶); molecular graphics: *PLATON* (Spek, 2009 ▶); software used to prepare material for publication: *SHELXL97* (Sheldrick, 2008 ▶).

## Supplementary Material

Crystal structure: contains datablocks I, global. DOI: 10.1107/S1600536810012067/ci5072sup1.cif

Structure factors: contains datablocks I. DOI: 10.1107/S1600536810012067/ci5072Isup2.hkl

Additional supplementary materials:  crystallographic information; 3D view; checkCIF report

## Figures and Tables

**Table 1:** Hydrogen-bond geometry (Å, °)

*D*—H⋯*A*	*D*—H	H⋯*A*	*D*⋯*A*	*D*—H⋯*A*
N1—H1*N*⋯O1^i^	0.81 (3)	2.09 (3)	2.902 (4)	172 (5)

Symmetry code: (i) 
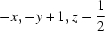
.

**Fig. 1.**
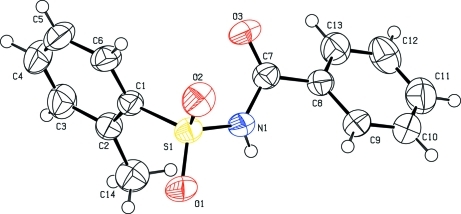
Molecular structure of the title compound, showing the atom-labelling scheme. Displacement ellipsoids are drawn at the 50% probability level.

**Fig. 2.**
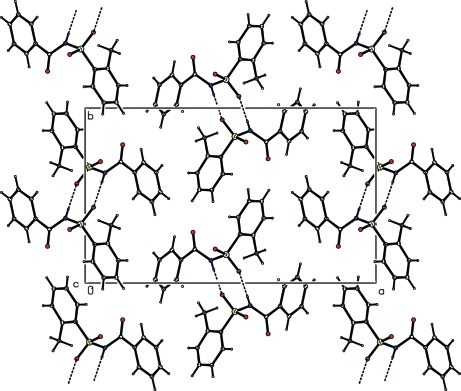
Molecular packing in the title compound. Hydrogen bonds are shown as dashed lines.

## supplementary crystallographic information

### Comment

As a part of studying the effect of ring and the side chain substituents on the crystal structures of *N*-aromatic sulfonamides (Gowda *et al.*, 2009, 2010; Suchetan *et al.*, 2010), the structure of *N*-(benzoyl)2-methylbenzenesulfonamide, (I), has been determined.

The conformation of the N–H bond in the C—SO_2_—NH—C(O) segment is *anti* to the C═O bond (Fig.1), similar to those observed in *N*-(benzoyl)benzenesulfonamide (II) (Gowda *et al.*, 2009), *N*-(benzoyl)2-chlorobenzenesulfonamide (III) (Gowda *et al.*, 2010) and *N*-(benzoyl)4-methylbenzenesulfonamide (IV) (Suchetan *et al.*, 2010).

The sulfonyl-bound tolyl and benzoyl groups are twisted about the S—N bond with a C1—S1—N1—C7 torsional angle of 68.8 (4)°, compared to 66.7 (2)° in (III) and 73.2 (2)° in (IV). The dihedral angle between the sulfonyl and the benzoyl benzene rings is 73.9 (1)°, compared to the values of 80.3(0.1) in (II), 73.3 (1)° in (III) and 79.4 (1)° in (IV).

The packing of molecules linked by of N—H···O(S) hydrogen bonds (Table 1) is shown in Fig. 2.

### Experimental

The title compound was prepared by refluxing a mixture of benzoic acid (0.02 mol), 2-methylbenzenesulfonamide (0.02 mol) and excess phosphorous oxy chloride for 3 h on a water bath. The resultant mixture was cooled and poured into crushed ice. The solid, *N*-(benzoyl)-2-methyl-benzenesulfonamide, obtained was filtered, washed thoroughly with water and then dissolved in a sodium bicarbonate solution. The compound was later reprecipitated by acidifying the filtered solution with dilute HCl. It was filtered, dried and recrystallized. Rod like colourless single crystals of the title compound used in X-ray diffraction studies were obtained by slow evaporation of its toluene solution at room temperature.

### Refinement

The H atom of the NH group was located in a difference map and refined with a N–H distance restraint of 0.86 (3) %A. The other H atoms were positioned with idealized geometry using a riding model with C–H = 0.93–0.96 Å. All H atoms were refined with isotropic displacement parameters (set to 1.2 times of the *U*_eq_ of the parent atom).

### Crystal data

**Table:**

C_14_H_13_NO_3_S	*F*(000) = 576
*M**_r_* = 275.31	*D*_x_ = 1.380 Mg m^−^^3^
Orthorhombic, *P**n**a*2_1_	Mo *K*α radiation, λ = 0.71073 Å
Hall symbol: P 2c -2n	Cell parameters from 2401 reflections
*a* = 19.772 (2) Å	θ = 3.1–27.9°
*b* = 11.894 (1) Å	µ = 0.25 mm^−^^1^
*c* = 5.6368 (5) Å	*T* = 299 K
*V* = 1325.6 (2) Å^3^	Rod, colourless
*Z* = 4	0.30 × 0.18 × 0.04 mm

### Data collection

**Table:**

Oxford Diffraction Xcalibur diffractometer with a Sapphire CCD detector	2028 independent reflections
Radiation source: fine-focus sealed tube	1760 reflections with *I* > 2σ(*I*)
graphite	*R*_int_ = 0.029
Rotation method data acquisition using ω and φ scans	θ_max_ = 26.4°, θ_min_ = 3.4°
Absorption correction: multi-scan (*CrysAlis RED*; Oxford Diffraction, 2009)	*h* = −24→24
*T*_min_ = 0.930, *T*_max_ = 0.990	*k* = −14→11
4891 measured reflections	*l* = −5→7

### Refinement

**Table:**

Refinement on *F*^2^	Secondary atom site location: difference Fourier map
Least-squares matrix: full	Hydrogen site location: inferred from neighbouring sites
*R*[*F*^2^ > 2σ(*F*^2^)] = 0.053	H atoms treated by a mixture of independent and constrained refinement
*wR*(*F*^2^) = 0.132	*w* = 1/[σ^2^(*F*_o_^2^) + (0.0676*P*)^2^ + 0.4459*P*] where *P* = (*F*_o_^2^ + 2*F*_c_^2^)/3
*S* = 1.18	(Δ/σ)_max_ = 0.001
2028 reflections	Δρ_max_ = 0.52 e Å^−^^3^
176 parameters	Δρ_min_ = −0.38 e Å^−^^3^
2 restraints	Absolute structure: Flack (1983), 541 Friedel pairs
Primary atom site location: structure-invariant direct methods	Flack parameter: −0.11 (15)

### Special details

**Table:**

Geometry. All esds (except the esd in the dihedral angle between two l.s. planes) are estimated using the full covariance matrix. The cell esds are taken into account individually in the estimation of esds in distances, angles and torsion angles; correlations between esds in cell parameters are only used when they are defined by crystal symmetry. An approximate (isotropic) treatment of cell esds is used for estimating esds involving l.s. planes.
Refinement. Refinement of *F*^2^ against ALL reflections. The weighted *R*-factor wR and goodness of fit *S* are based on *F*^2^, conventional *R*-factors *R* are based on *F*, with *F* set to zero for negative *F*^2^. The threshold expression of *F*^2^ > σ(*F*^2^) is used only for calculating *R*-factors(gt) etc. and is not relevant to the choice of reflections for refinement. *R*-factors based on *F*^2^ are statistically about twice as large as those based on *F*, and *R*- factors based on ALL data will be even larger.

### Fractional atomic coordinates and isotropic or equivalent isotropic displacement parameters (Å^2^)

**Table:**

	*x*	*y*	*z*	*U*_iso_*/*U*_eq_	
S1	0.01516 (5)	0.66278 (7)	0.7707 (2)	0.0435 (3)	
O1	−0.02885 (13)	0.56674 (19)	0.7833 (7)	0.0557 (8)	
O2	0.05286 (16)	0.6940 (3)	0.9718 (6)	0.0641 (9)	
O3	0.12916 (15)	0.7901 (2)	0.5428 (7)	0.0668 (9)	
N1	0.06761 (16)	0.6295 (3)	0.5573 (7)	0.0452 (9)	
H1N	0.058 (2)	0.578 (3)	0.469 (7)	0.054*	
C1	−0.03320 (19)	0.7795 (3)	0.6767 (8)	0.0419 (9)	
C2	−0.07765 (19)	0.7733 (3)	0.4882 (8)	0.0462 (9)	
C3	−0.1141 (2)	0.8708 (4)	0.4372 (10)	0.0660 (14)	
H3	−0.1439	0.8710	0.3095	0.079*	
C4	−0.1069 (3)	0.9667 (4)	0.5716 (12)	0.0715 (15)	
H4	−0.1320	1.0304	0.5338	0.086*	
C5	−0.0643 (3)	0.9694 (3)	0.7562 (12)	0.0686 (14)	
H5	−0.0604	1.0344	0.8469	0.082*	
C6	−0.0263 (2)	0.8761 (3)	0.8122 (9)	0.0542 (12)	
H6	0.0037	0.8779	0.9393	0.065*	
C7	0.12242 (19)	0.6934 (3)	0.4833 (8)	0.0467 (10)	
C8	0.17044 (19)	0.6327 (3)	0.3244 (8)	0.0458 (10)	
C9	0.1851 (2)	0.5197 (3)	0.3616 (10)	0.0549 (11)	
H9	0.1652	0.4808	0.4864	0.066*	
C10	0.2297 (2)	0.4660 (4)	0.2110 (12)	0.0692 (15)	
H10	0.2402	0.3908	0.2376	0.083*	
C11	0.2586 (2)	0.5202 (4)	0.0250 (13)	0.0754 (15)	
H11	0.2876	0.4821	−0.0771	0.091*	
C12	0.2444 (2)	0.6331 (5)	−0.0109 (11)	0.0715 (14)	
H12	0.2641	0.6713	−0.1371	0.086*	
C13	0.2012 (2)	0.6883 (4)	0.1397 (10)	0.0616 (12)	
H13	0.1926	0.7644	0.1166	0.074*	
C14	−0.0893 (3)	0.6700 (4)	0.3393 (9)	0.0638 (14)	
H14A	−0.1102	0.6128	0.4342	0.077*	
H14B	−0.0467	0.6428	0.2804	0.077*	
H14C	−0.1182	0.6884	0.2082	0.077*	

### Atomic displacement parameters (Å^2^)

**Table:**

	*U*^11^	*U*^22^	*U*^33^	*U*^12^	*U*^13^	*U*^23^
S1	0.0501 (5)	0.0380 (4)	0.0425 (5)	−0.0008 (4)	−0.0026 (6)	0.0032 (5)
O1	0.0651 (17)	0.0372 (12)	0.065 (2)	−0.0059 (10)	0.005 (2)	0.0136 (16)
O2	0.0712 (19)	0.0661 (17)	0.055 (2)	0.0070 (16)	−0.0160 (17)	0.0020 (17)
O3	0.0731 (19)	0.0327 (12)	0.095 (3)	−0.0069 (13)	0.011 (2)	−0.0074 (16)
N1	0.0459 (18)	0.0366 (15)	0.053 (2)	−0.0042 (13)	0.0025 (18)	−0.0071 (16)
C1	0.0450 (19)	0.0377 (18)	0.043 (2)	−0.0016 (14)	0.0079 (18)	0.0020 (16)
C2	0.046 (2)	0.045 (2)	0.048 (3)	0.0009 (16)	0.002 (2)	0.0005 (19)
C3	0.059 (3)	0.065 (3)	0.075 (4)	0.013 (2)	−0.006 (3)	0.011 (3)
C4	0.069 (3)	0.050 (2)	0.095 (5)	0.017 (2)	0.004 (3)	0.006 (3)
C5	0.081 (3)	0.0373 (19)	0.087 (4)	0.0009 (19)	0.026 (4)	−0.009 (3)
C6	0.061 (3)	0.0428 (18)	0.058 (4)	−0.0068 (17)	0.004 (2)	−0.010 (2)
C7	0.047 (2)	0.0415 (19)	0.052 (3)	−0.0001 (16)	−0.002 (2)	0.0041 (19)
C8	0.043 (2)	0.0416 (18)	0.052 (3)	−0.0011 (15)	−0.003 (2)	0.0018 (17)
C9	0.052 (2)	0.0411 (19)	0.072 (3)	0.0015 (17)	0.000 (2)	0.0031 (19)
C10	0.056 (2)	0.053 (2)	0.098 (5)	0.0040 (19)	0.007 (3)	−0.009 (3)
C11	0.060 (3)	0.085 (3)	0.082 (4)	0.006 (3)	−0.002 (3)	−0.022 (4)
C12	0.057 (3)	0.095 (3)	0.063 (3)	−0.001 (3)	0.013 (3)	0.013 (3)
C13	0.056 (3)	0.060 (2)	0.069 (3)	0.006 (2)	−0.001 (3)	0.015 (2)
C14	0.070 (3)	0.067 (3)	0.055 (3)	−0.001 (2)	−0.012 (2)	−0.008 (2)

### Geometric parameters (Å, °)

**Table:**

S1—O2	1.407 (3)	C6—H6	0.93
S1—O1	1.438 (2)	C7—C8	1.491 (6)
S1—N1	1.637 (4)	C8—C13	1.375 (6)
S1—C1	1.767 (4)	C8—C9	1.392 (5)
O3—C7	1.206 (5)	C9—C10	1.380 (7)
N1—C7	1.387 (5)	C9—H9	0.93
N1—H1N	0.81 (3)	C10—C11	1.357 (9)
C1—C2	1.381 (6)	C10—H10	0.93
C1—C6	1.386 (6)	C11—C12	1.387 (7)
C2—C3	1.395 (6)	C11—H11	0.93
C2—C14	1.505 (6)	C12—C13	1.371 (7)
C3—C4	1.377 (7)	C12—H12	0.93
C3—H3	0.93	C13—H13	0.93
C4—C5	1.339 (8)	C14—H14A	0.96
C4—H4	0.93	C14—H14B	0.96
C5—C6	1.377 (6)	C14—H14C	0.96
C5—H5	0.93		
			
O2—S1—O1	119.4 (2)	O3—C7—N1	121.7 (4)
O2—S1—N1	108.70 (19)	O3—C7—C8	123.9 (4)
O1—S1—N1	103.17 (18)	N1—C7—C8	114.4 (3)
O2—S1—C1	108.7 (2)	C13—C8—C9	119.1 (4)
O1—S1—C1	108.14 (17)	C13—C8—C7	120.3 (4)
N1—S1—C1	108.21 (19)	C9—C8—C7	120.6 (4)
C7—N1—S1	125.7 (3)	C10—C9—C8	119.2 (4)
C7—N1—H1N	115 (3)	C10—C9—H9	120.4
S1—N1—H1N	119 (3)	C8—C9—H9	120.4
C2—C1—C6	122.1 (4)	C11—C10—C9	121.6 (4)
C2—C1—S1	122.2 (3)	C11—C10—H10	119.2
C6—C1—S1	115.6 (3)	C9—C10—H10	119.2
C1—C2—C3	116.3 (4)	C10—C11—C12	119.2 (5)
C1—C2—C14	124.7 (4)	C10—C11—H11	120.4
C3—C2—C14	119.0 (4)	C12—C11—H11	120.4
C4—C3—C2	121.5 (5)	C13—C12—C11	120.0 (5)
C4—C3—H3	119.2	C13—C12—H12	120.0
C2—C3—H3	119.2	C11—C12—H12	120.0
C5—C4—C3	120.8 (4)	C12—C13—C8	120.9 (4)
C5—C4—H4	119.6	C12—C13—H13	119.5
C3—C4—H4	119.6	C8—C13—H13	119.5
C4—C5—C6	120.2 (5)	C2—C14—H14A	109.5
C4—C5—H5	119.9	C2—C14—H14B	109.5
C6—C5—H5	119.9	H14A—C14—H14B	109.5
C5—C6—C1	119.2 (5)	C2—C14—H14C	109.5
C5—C6—H6	120.4	H14A—C14—H14C	109.5
C1—C6—H6	120.4	H14B—C14—H14C	109.5
			
O2—S1—N1—C7	−49.1 (4)	C4—C5—C6—C1	−0.6 (7)
O1—S1—N1—C7	−176.8 (3)	C2—C1—C6—C5	−0.7 (6)
C1—S1—N1—C7	68.8 (4)	S1—C1—C6—C5	−177.4 (3)
O2—S1—C1—C2	−177.3 (3)	S1—N1—C7—O3	−13.8 (6)
O1—S1—C1—C2	−46.3 (4)	S1—N1—C7—C8	167.0 (3)
N1—S1—C1—C2	64.8 (3)	O3—C7—C8—C13	−36.2 (7)
O2—S1—C1—C6	−0.5 (4)	N1—C7—C8—C13	143.0 (4)
O1—S1—C1—C6	130.5 (3)	O3—C7—C8—C9	143.4 (5)
N1—S1—C1—C6	−118.4 (3)	N1—C7—C8—C9	−37.5 (6)
C6—C1—C2—C3	1.6 (6)	C13—C8—C9—C10	−0.6 (6)
S1—C1—C2—C3	178.2 (3)	C7—C8—C9—C10	179.9 (4)
C6—C1—C2—C14	−177.9 (4)	C8—C9—C10—C11	−1.2 (7)
S1—C1—C2—C14	−1.3 (6)	C9—C10—C11—C12	1.7 (8)
C1—C2—C3—C4	−1.4 (7)	C10—C11—C12—C13	−0.4 (8)
C14—C2—C3—C4	178.1 (5)	C11—C12—C13—C8	−1.3 (8)
C2—C3—C4—C5	0.2 (8)	C9—C8—C13—C12	1.8 (7)
C3—C4—C5—C6	0.9 (8)	C7—C8—C13—C12	−178.6 (4)

### Hydrogen-bond geometry (Å, °)

**Table:**

*D*—H···*A*	*D*—H	H···*A*	*D*···*A*	*D*—H···*A*
N1—H1N···O1^i^	0.81 (3)	2.09 (3)	2.902 (4)	172 (5)

Symmetry codes: (i) −*x*, −*y*+1, *z*−1/2.
